# The genome sequence of the Flame Carpet moth, *Xanthorhoe designata *(Hufnagel, 1767) (Lepidoptera: Geometridae)

**DOI:** 10.12688/wellcomeopenres.23757.2

**Published:** 2025-10-01

**Authors:** Gavin R. Broad, Laura Sivess, Steph Holt, Natural History Museum Genome Acquisition Lab, Darwin Tree of Life Barcoding collective, Wellcome Sanger Institute Tree of Life Management, Samples and Laboratory team, Wellcome Sanger Institute Scientific Operations: Sequencing Operations, Wellcome Sanger Institute Tree of Life Core Informatics team, Tree of Life Core Informatics collective, Darwin Tree of Life Consortium

**Affiliations:** 1 Natural History Museum, London, England, UK

**Keywords:** Xanthorhoe designata, Flame Carpet, genome sequence, chromosomal, Lepidoptera

## Abstract

We present a genome assembly from a female *Xanthorhoe designata* (Flame Carpet; Arthropoda; Insecta; Lepidoptera; Geometridae). The genome sequence has a total length of 351.47 megabases. Most of the assembly (99.45%) is scaffolded into 31 chromosomal pseudomolecules, including the W and Z sex chromosomes. The mitochondrial genome has also been assembled and is 17.55 kilobases in length. Gene annotation of this assembly on Ensembl identified 12,291 protein-coding genes. This assembly was generated as part of the Darwin Tree of Life project, which produces reference genomes for eukaryotic species found in Britain and Ireland.

## Species taxonomy

Eukaryota; Opisthokonta; Metazoa; Eumetazoa; Bilateria; Protostomia; Ecdysozoa; Panarthropoda; Arthropoda; Mandibulata; Pancrustacea; Hexapoda; Insecta; Dicondylia; Pterygota; Neoptera; Endopterygota; Amphiesmenoptera; Lepidoptera; Glossata; Neolepidoptera; Heteroneura; Ditrysia; Obtectomera; Geometroidea; Geometridae; Larentiinae; *Xanthorhoe*; *Xanthorhoe designata* (Hufnagel, 1767) (NCBI:txid934901)

## Background

*Xanthorhoe designata* owes its English name, Flame Carpet, to the pale reddish (or purplish), black-lined central band across the otherwise mostly grey fore wings. The species name, from the Latin *designo*, ‘to define’, probably also references this distinctive identification feature ( Emmet, 1991). The adult is easily recognised but the larva is very difficult to distinguish from other *Xanthorhoe* carpets, such as *X. spadicearia* and *X. ferrugata* and should be reared to confirm the identity ( Henwood *et al.*, 2020). Consequently, the foodplants of *X. designata* are poorly known; Waring *et al.* (2017) report that larvae readily eat Brassicaceae in captivity and it can be found hiding in withered leaves of Mugwort ( *Artemisia vulgaris*, family Asteraceae) ( Henwood *et al.*, 2020).

Many habitats are home to *X. designata* and it is a common and widespread species across Britain, easily light-trapped in gardens, woodland, uplands, etc. In recent decades, *X. designata* has become significantly more abundant and widespread and the spring generation is on the wing earlier in the year ( Randle *et al.*, 2019). There are two generations per year in Britain, peaking in May/June and then August. It has a wide range across the northern Palaearctic and is regarded as a typical species of the taiga ( Kullberg *et al.*, 2019). Further South, *X. designata* is less abundant, for example only recently being recorded as new to Portugal ( Marabuto, 2022).

The genome of *X. designata* adds to a growing number of available *Xanthorhoe* genomes (e.g., Boyes *et al.*, 2024) and will facilitate comparative genomics of a group of geometrid moths of contrasting fortunes in the modern landscape. This assembly was generated as part of the Darwin Tree of Life Project, which aims to generate high-quality reference genomes for all named eukaryotic species in Britain and Ireland to support research, conservation, and the sustainable use of biodiversity ( Blaxter *et al*., 2022).

## Genome sequence report

### Sequencing data

**Figure 1.**
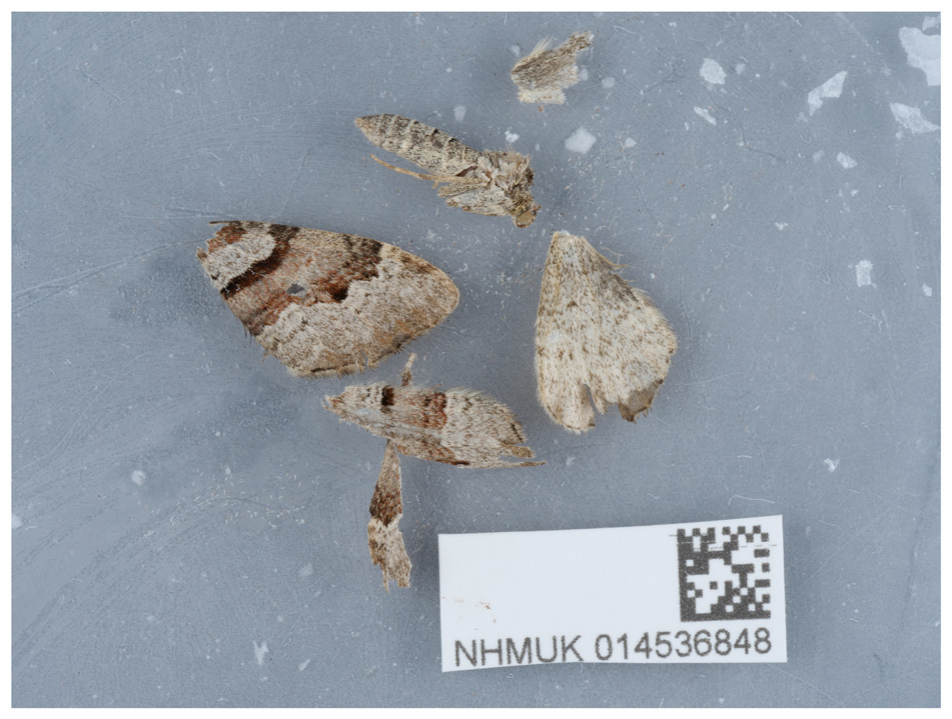
Photograph of the *Xanthorhoe designata* (ilXanDesi1) specimen used for genome sequencing.

The genome of a specimen of *Xanthorhoe designata* ( Figure 1) was sequenced using Pacific Biosciences single-molecule HiFi long reads, generating 22.74 Gb from 2.16 million reads. GenomeScope analysis of the PacBio HiFi data estimated the haploid genome size at 338.65 Mb, with a heterozygosity of 1.71% and repeat content of 27.20%. These values provide an initial assessment of genome complexity and the challenges anticipated during assembly. Based on this estimated genome size, the sequencing data provided approximately 62× coverage of the genome. Hi-C sequencing 146.87 Gb from 972.63 million reads. Table 1 summarises the specimen and sequencing information.

**Table 1.** Specimen and sequencing data for *Xanthorhoe designata*.

Project information			
**Study title**	Xanthorhoe designata		
**Umbrella BioProject**	PRJEB63491		
**Species**	*Xanthorhoe designata*		
**BioSample**	SAMEA112222336		
**NCBI taxonomy ID**	934901		
Specimen information			
**Technology**	**ToLID**	**BioSample accession**	**Organism part**
**PacBio long read sequencing**	ilXanDesi1	SAMEA112222422	head and thorax
**Hi-C sequencing**	ilXanDesi1	SAMEA112222422	head and thorax
Sequencing information			
**Platform**	**Run accession**	**Read count**	**Base count (Gb)**
**Hi-C Illumina NovaSeq 6000**	ERR11606330	9.73e+08	146.87
**PacBio Sequel IIe**	ERR11641054	2.16e+06	22.74

### Assembly statistics

The primary haplotype was assembled, and contigs corresponding to an alternate haplotype were also deposited in INSDC databases. The assembly was improved by manual curation, which corrected 33 misjoins or missing joins and removed one haplotypic duplication. These interventions decreased the scaffold count by 18.58% and increased the scaffold N50 by 4.62%. The final assembly has a total length of 351.47 Mb in 91 scaffolds, with 77 gaps, and a scaffold N50 of 12.72 Mb ( Table 2).

**Table 2.** Genome assembly data for *Xanthorhoe designata*.

Genome assembly		
Assembly name	ilXanDesi1.1	
Assembly accession	GCA_963582015.1	
*Alternate haplotype accession*	*GCA_963583385.1*	
Assembly level for primary assembly	chromosome	
Span (Mb)	351.47	
Number of contigs	168	
Number of scaffolds	91	
Longest scaffold (Mb)	17.6	
Assembly metrics	Measure	*Benchmark*
Contig N50 length	6.19 Mb	≥ *1 Mb*
Scaffold N50 length	12.72 Mb	= *chromosome N50*
Consensus quality (QV)	Primary: 66.4; alternate: 68.3; combined 67.2	≥ *40*
*k*-mer completeness	Primary: 71.46%; alternate: 71.27%; combined: 98.81%	≥ *95%*
BUSCO v 5.8.2 *	Using lepidoptera_odb10:C:97.8%[S:97.3%,D:0.5%], F:0.4%,M:1.8%,n:5,286 Using lepidoptera_odb12: C:96.9%[S:96.6%,D:0.3%], F:1.6%,M:1.5%,n:5760	*S* > *90%*; *D* < *5%*
Percentage of assembly mapped to chromosomes	99.44%	≥ *90%*
Sex chromosomes	W and Z	*localised homologous* *pairs*
Organelles	Mitochondrial genome: 17.55 kb	*complete single alleles*
Genome annotation of assembly GCA_963582015.1 at Ensembl		
Number of protein-coding genes	12,291	
Number of non-coding genes	1,683	
Number of gene transcripts	21,986	

* [S = single copy, D = duplicated], F = fragmented, M = missing, n = number of orthologues in comparison.

**Figure 2.**
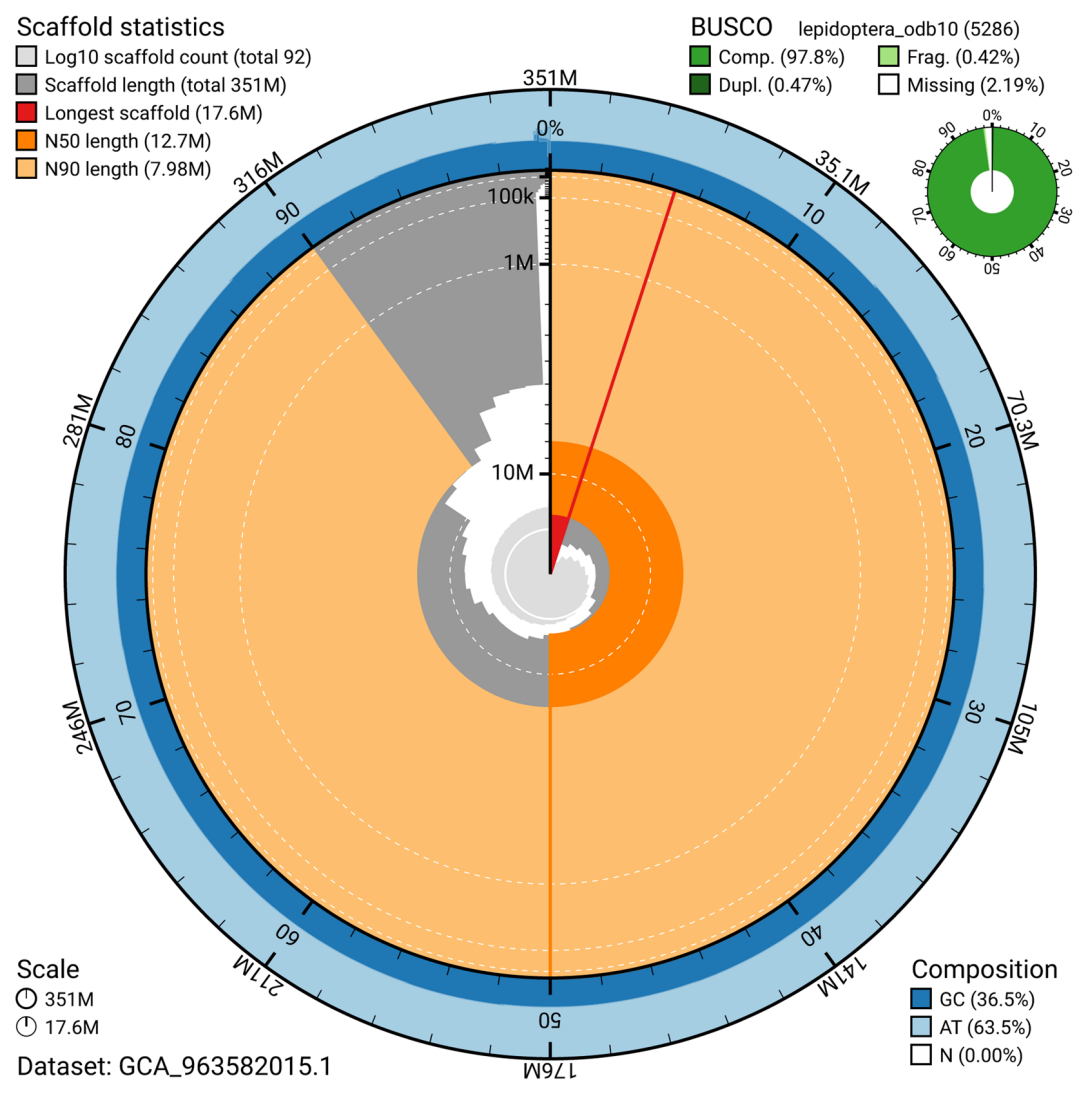
Genome assembly of *Xanthorhoe designata*, ilXanDesi1.1: metrics. The BlobToolKit snail plot provides an overview of assembly metrics and BUSCO gene completeness. The circumference represents the length of the whole genome sequence, and the main plot is divided into 1,000 bins around the circumference. The outermost blue tracks display the distribution of GC, AT, and N percentages across the bins. Scaffolds are arranged clockwise from longest to shortest and are depicted in dark grey. The longest scaffold is indicated by the red arc, and the deeper orange and pale orange arcs represent the N50 and N90 lengths. A light grey spiral at the centre shows the cumulative scaffold count on a logarithmic scale. A summary of complete, fragmented, duplicated, and missing BUSCO genes in the lepidoptera_odb10 set is presented at the top right. An interactive version of this figure is available at https://blobtoolkit.genomehubs.org/view/GCA_963582015.1/dataset/GCA_963582015.1/snail.

**Figure 3.**
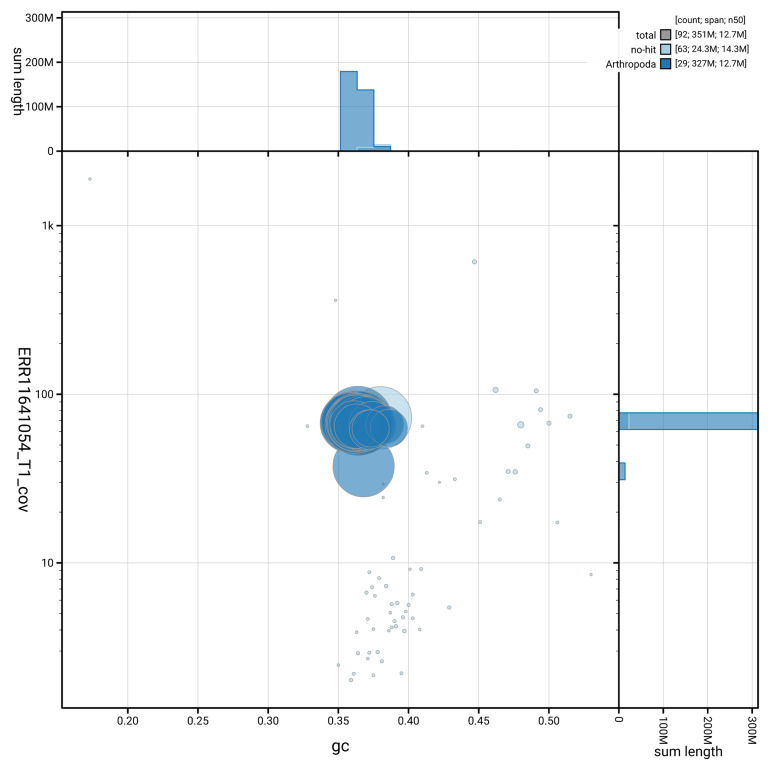
Genome assembly of *Xanthorhoe designata*, ilXanDesi1.1: BlobToolKit GC-coverage plot. Blob plot showing sequence coverage (vertical axis) and GC content (horizontal axis). The circles represent scaffolds, with the size proportional to scaffold length and the colour representing phylum membership. The histograms along the axes display the total length of sequences distributed across different levels of coverage and GC content. An interactive version of this figure is available at https://blobtoolkit.genomehubs.org/view/GCA_963582015.1/blob.

**Figure 4.**
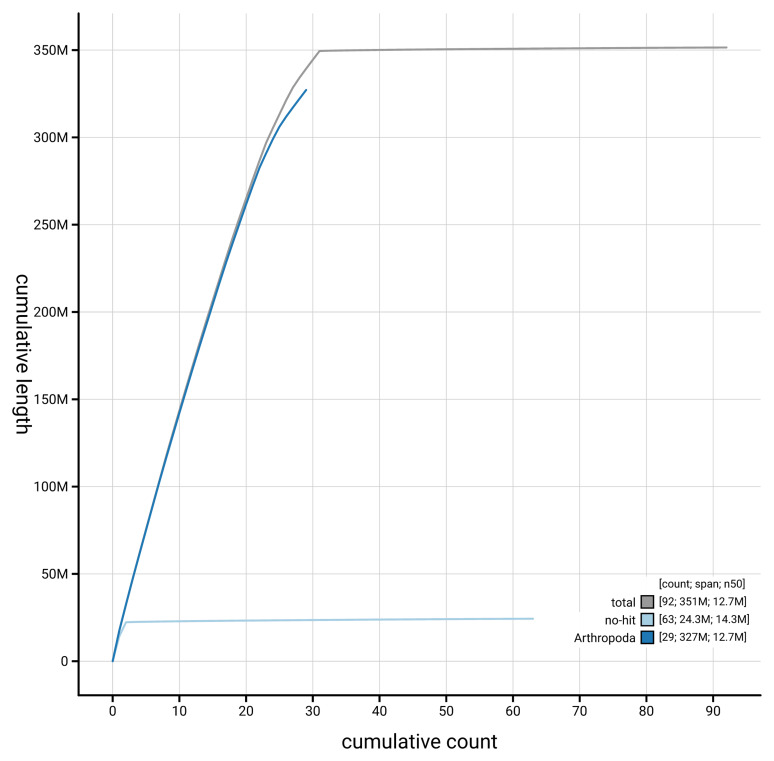
Genome assembly of *Xanthorhoe designata,* ilXanDesi1.1: BlobToolKit cumulative sequence plot. The grey line shows cumulative length for all scaffolds. Coloured lines show cumulative lengths of scaffolds assigned to each phylum using the buscogenes taxrule. An interactive version of this figure is available at https://blobtoolkit.genomehubs.org/view/GCA_963582015.1/dataset/GCA_963582015.1/cumulative.

The snail plot in Figure 2 provides a summary of the assembly statistics, indicating the distribution of scaffold lengths and other assembly metrics. Figure 3 shows the distribution of scaffolds by GC proportion and coverage. Figure 4 presents a cumulative assembly plot, with separate curves representing different scaffold subsets assigned to various phyla, illustrating the completeness of the assembly.

**Figure 5.**
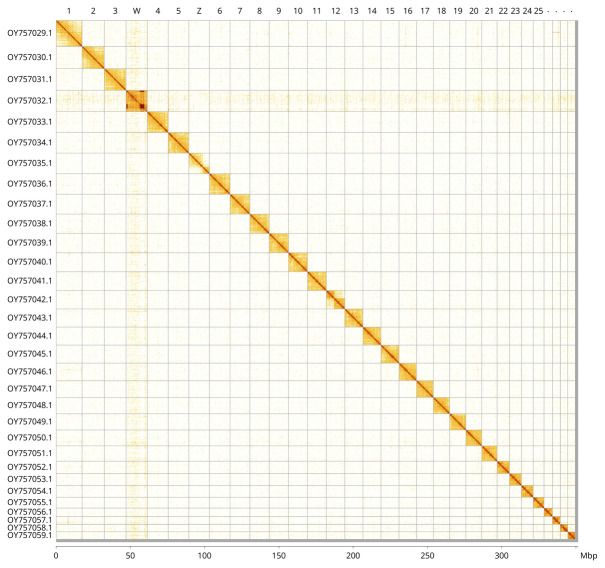
Genome assembly of *Xanthorhoe designata*: Hi-C contact map of the ilXanDesi1.1 assembly, visualised using PretextView and PretextSnapshot. Assembled chromosomes are shown in order of size and labelled along the axes, with a megabase scale shown below. An interactive version of this figure may be viewed in HiGlass at https://genome-note-higlass.tol.sanger.ac.uk/l/?d=O79C2EW9Sduip1ZM9j-kCQ.

Most of the assembly sequence (99.44%) was assigned to 31 chromosomal-level scaffolds, representing 29 autosomes and the W and Z sex chromosomes. These chromosome-level scaffolds, confirmed by Hi-C data, are named according to size ( Figure 5; Table 3).

**Table 3.** Chromosomal pseudomolecules in the genome assembly of *Xanthorhoe designata*, ilXanDesi1.

INSDC accession	Name	Length (Mb)	GC%
OY757029.1	1	17.6	36.5
OY757030.1	2	14.96	36.5
OY757031.1	3	14.63	36.5
OY757033.1	4	14.02	36.5
OY757034.1	5	13.9	36
OY757036.1	6	13.82	36.5
OY757037.1	7	13.35	36
OY757038.1	8	13.02	36
OY757039.1	9	12.99	36
OY757040.1	10	12.79	36
OY757041.1	11	12.72	36
OY757042.1	12	12.42	36.5
OY757043.1	13	12.16	36
OY757044.1	14	12.15	36
OY757045.1	15	12.13	36
OY757046.1	16	11.82	36.5
OY757047.1	17	11.27	36
OY757048.1	18	11.01	37
OY757049.1	19	10.97	36.5
OY757050.1	20	10.66	36.5
OY757051.1	21	10.27	37
OY757052.1	22	8.35	36
OY757053.1	23	8.01	37.5
OY757054.1	24	7.98	36.5
OY757055.1	25	7.24	36.5
OY757056.1	26	5.69	37
OY757057.1	27	5.2	38.5
OY757058.1	28	5.08	38.5
OY757059.1	29	5.02	37.5
OY757032.1	W	14.33	38
OY757035.1	Z	13.89	37
OY757060.1	MT	0.02	17.5

The mitochondrial genome was also assembled. This sequence is included as a contig in the multifasta file of the genome submission and as a standalone record in GenBank.

### Assembly quality metrics

The estimated Quality Value (QV) and *k*-mer completeness metrics, along with BUSCO completeness scores, were calculated for each haplotype and the combined assembly. The QV reflects the base-level accuracy of the assembly, while *k*-mer completeness indicates the proportion of expected *k*-mers identified in the assembly. BUSCO scores provide a measure of completeness based on benchmarking universal single-copy orthologues.

The primary haplotype has a QV of 66.4, and the combined primary and alternate assemblies achieve an estimated QV of 67.2. The *k*-mer completeness for the primary haplotype is 71.46%, and for the alternate haplotype it is 71.27%. The combined primary and alternate assemblies achieve a *k*-mer completeness of 98.81%.

BUSCO v5.8.2 analysis using the lepidoptera_odb10 reference set ( *n* = 5,286) indicated a completeness score of 97.8% (single = 97.3%, duplicated = 0.5%). In comparison, BUSCO v5.8.2 analysis using the lepidoptera_odb12 reference set ( *n* = 5,760) gave a slightly lower completeness score of 96.9% (single = 96.6%, duplicated = 0.3%).

Table 2 provides assembly metric benchmarks adapted from Rhie *et al.* (2021) and the Earth BioGenome Project (EBP) Report on Assembly Standards September 2024. The assembly achieves the EBP quality code of **6.C.Q66**.

## Genome annotation report

The *Xanthorhoe designata* genome assembly (GCA_963582015.1) was annotated at the European Bioinformatics Institute (EBI) on Ensembl Rapid Release. The resulting annotation includes 21,986 transcribed mRNAs from 12,291 protein-coding and 1,683 non-coding genes. The average transcript length is 13,079.22, with an average of 1.57 coding transcripts per gene and 7.35 exons per transcript. For further information about the annotation, please refer to the annotation page on Ensembl.

## Methods

### Sample acquisition and DNA barcoding

A female *Xanthorhoe designata* (specimen ID NHMUK014536848, ToLID ilXanDesi1) was collected from Gilbert White's House, Selborne, England, United Kingdom (latitude 51.09, longitude -–0.94) on 2021-06-10, using a light trap. The specimen was collected by Laura Sivess, Steph Holt and Gavin Broad (Natural History Museum), identified by Gavin Broad and preserved by dry freezing (–80 °C).

The initial identification by morphology was verified by an additional DNA barcoding process according to the framework developed by Twyford *et al*. (2024). A small sample was dissected from the specimen and stored in ethanol, while the remaining parts were shipped on dry ice to the Wellcome Sanger Institute (WSI) ( Pereira *et al.*, 2022). The tissue was lysed, the COI marker region was amplified by PCR, and amplicons were sequenced and compared to the BOLD database, confirming the species identification ( Crowley *et al.*, 2023). Following whole genome sequence generation, the relevant DNA barcode region was also used alongside the initial barcoding data for sample tracking at the WSI ( Twyford *et al.*, 2024). The standard operating procedures for Darwin Tree of Life barcoding have been deposited on protocols.io ( Beasley *et al.*, 2023).

Metadata collection for samples adhered to the Darwin Tree of Life project standards described by Lawniczak *et al*. (2022).

### Nucleic acid extraction

The workflow for high molecular weight (HMW) DNA extraction at the Wellcome Sanger Institute (WSI) Tree of Life Core Laboratory includes a sequence of procedures: sample preparation and homogenisation, DNA extraction, fragmentation and purification. Detailed protocols are available on protocols.io ( Denton *et al.*, 2023b). The ilXanDesi1 sample was prepared for DNA extraction by weighing and dissecting it on dry ice ( Jay *et al.*, 2023). Tissue from the head and thorax was homogenised using a PowerMasher II tissue disruptor ( Denton *et al.*, 2023a).

HMW DNA was extracted in the WSI Scientific Operations core using the Automated MagAttract v2 protocol ( Oatley *et al.*, 2023). The DNA was sheared into an average fragment size of 12–20 kb in a Megaruptor 3 system ( Bates *et al.*, 2023). Sheared DNA was purified by solid-phase reversible immobilisation, using AMPure PB beads to eliminate shorter fragments and concentrate the DNA ( Strickland *et al.*, 2023). The concentration of the sheared and purified DNA was assessed using a Nanodrop spectrophotometer and Qubit Fluorometer using the Qubit dsDNA High Sensitivity Assay kit. Fragment size distribution was evaluated by running the sample on the FemtoPulse system.

### Hi-C sample preparation

Tissue from the head and thorax of the ilXanDesi1 sample was processed for Hi-C sequencing at the WSI Scientific Operations core, using the Arima-HiC v2 kit. In brief, 20–50 mg of frozen tissue (stored at –80 °C) was fixed, and the DNA crosslinked using a TC buffer with 22% formaldehyde concentration. After crosslinking, the tissue was homogenised using the Diagnocine Power Masher-II and BioMasher-II tubes and pestles. Following the Arima-HiC v2 kit manufacturer's instructions, crosslinked DNA was digested using a restriction enzyme master mix. The 5’-overhangs were filled in and labelled with biotinylated nucleotides and proximally ligated. An overnight incubation was carried out for enzymes to digest remaining proteins and for crosslinks to reverse. A clean up was performed with SPRIselect beads prior to library preparation. Additionally, the biotinylation percentage was estimated using the Qubit Fluorometer v4.0 (Thermo Fisher Scientific) and Qubit HS Assay Kit and Arima-HiC v2 QC beads.

### Library preparation and sequencing

Library preparation and sequencing were performed at the WSI Scientific Operations core.

***PacBio HiFi***

At a minimum, samples were required to have an average fragment size exceeding 8 kb and a total mass over 400 ng to proceed to the low input SMRTbell Prep Kit 3.0 protocol (Pacific Biosciences, California, USA), depending on genome size and sequencing depth required. Libraries were prepared using the SMRTbell Prep Kit 3.0 (Pacific Biosciences, California, USA) as per the manufacturer's instructions. The kit includes the reagents required for end repair/A-tailing, adapter ligation, post-ligation SMRTbell bead cleanup, and nuclease treatment. Following the manufacturer’s instructions, size selection and clean up was carried out using diluted AMPure PB beads (Pacific Biosciences, California, USA). DNA concentration was quantified using the Qubit Fluorometer v4.0 (Thermo Fisher Scientific) with Qubit 1X dsDNA HS assay kit and the final library fragment size analysis was carried out using the Agilent Femto Pulse Automated Pulsed Field CE Instrument (Agilent Technologies) and gDNA 55kb BAC analysis kit.

Samples were sequenced using the Sequel IIe system (Pacific Biosciences, California, USA). The concentration of the library loaded onto the Sequel IIe was in the range 40–135 pM. The SMRT link software, a PacBio web-based end-to-end workflow manager, was used to set-up and monitor the run, as well as perform primary and secondary analysis of the data upon completion.

***Hi-C***

For Hi-C library preparation, DNA was fragmented using the Covaris E220 sonicator (Covaris) and size selected using SPRISelect beads to 400 to 600 bp. The DNA was then enriched using the Arima-HiC v2 kit Enrichment beads. Using the NEBNext Ultra II DNA Library Prep Kit (New England Biolabs) for end repair, A-tailing, and adapter ligation. This uses a custom protocol which resembles the standard NEBNext Ultra II DNA Library Prep protocol but where library preparation occurs while DNA is bound to the Enrichment beads. For library amplification, 10 to 16 PCR cycles were required, determined by the sample biotinylation percentage. The Hi-C sequencing was performed using paired-end sequencing with a read length of 150 bp on an Illumina NovaSeq 6000 instrument.

### Genome assembly, curation and evaluation

***Assembly***

Prior to assembly of the PacBio HiFi reads, a database of *k*-mer counts ( *k* = 31) was generated from the filtered reads using FastK. GenomeScope2 ( Ranallo-Benavidez *et al.*, 2020) was used to analyse the *k*-mer frequency distributions, providing estimates of genome size, heterozygosity, and repeat content.

The HiFi reads were first assembled using Hifiasm ( Cheng *et al.*, 2021) with the --primary option. Haplotypic duplications were identified and removed using purge_dups ( Guan *et al.*, 2020). The Hi-C reads were mapped to the primary contigs using bwa-mem2 ( Vasimuddin *et al.*, 2019). The contigs were further scaffolded using the provided Hi-C data ( Rao *et al.*, 2014) in YaHS ( Zhou *et al.*, 2023) using the --break option for handling potential misassemblies. The scaffolded assemblies were evaluated using Gfastats ( Formenti *et al.*, 2022), BUSCO ( Manni *et al.*, 2021) and MERQURY.FK ( Rhie *et al.*, 2020).

The mitochondrial genome was assembled using MitoHiFi ( Uliano-Silva *et al.*, 2023), which runs MitoFinder ( Allio *et al.*, 2020) and uses these annotations to select the final mitochondrial contig and to ensure the general quality of the sequence.

***Assembly curation***

The assembly was decontaminated using the Assembly Screen for Cobionts and Contaminants (ASCC) pipeline (article in preparation). Flat files and maps used in curation were generated in TreeVal ( Pointon *et al.*, 2023). Manual curation was primarily conducted using PretextView ( Harry, 2022), with additional insights provided by JBrowse2 ( Diesh *et al.*, 2023) and HiGlass ( Kerpedjiev *et al.*, 2018). Scaffolds were visually inspected and corrected as described by Howe *et al*. (2021). Any identified contamination, missed joins, and mis-joins were corrected, and duplicate sequences were tagged and removed. The curation process is documented at https://gitlab.com/wtsi-grit/rapid-curation (article in preparation).

***Assembly quality assessment***

The Merqury.FK tool ( Rhie *et al.*, 2020), run in a Singularity container ( Kurtzer *et al.*, 2017), was used to evaluate *k*-mer completeness and assembly quality for the primary and alternate haplotypes using the *k*-mer databases ( *k* = 31) that were computed prior to genome assembly. The analysis outputs included assembly QV scores and completeness statistics.

A Hi-C contact map was produced for the final version of the assembly. The Hi-C reads were aligned using bwa-mem2 ( Vasimuddin *et al.*, 2019) and the alignment files were combined using SAMtools ( Danecek *et al.*, 2021). The Hi-C alignments were converted into a contact map using BEDTools ( Quinlan & Hall, 2010) and the Cooler tool suite ( Abdennur & Mirny, 2020). The contact map is visualised in HiGlass ( Kerpedjiev *et al.*, 2018).

The blobtoolkit pipeline is a Nextflow port of the previous Snakemake Blobtoolkit pipeline ( Challis *et al.*, 2020). It aligns the PacBio reads in SAMtools and minimap2 ( Li, 2018) and generates coverage tracks for regions of fixed size. In parallel, it queries the GoaT database ( Challis *et al.*, 2023) to identify all matching BUSCO lineages to run BUSCO ( Manni *et al.*, 2021). For the three domain-level BUSCO lineages, the pipeline aligns the BUSCO genes to the UniProt Reference Proteomes database ( Bateman *et al.*, 2023) with DIAMOND blastp ( Buchfink *et al.*, 2021). The genome is also divided into chunks according to the density of the BUSCO genes from the closest taxonomic lineage, and each chunk is aligned to the UniProt Reference Proteomes database using DIAMOND blastx. Genome sequences without a hit are chunked using seqtk and aligned to the NT database with blastn ( Altschul *et al.*, 1990). The blobtools suite combines all these outputs into a blobdir for visualisation.

The blobtoolkit pipeline was developed using nf-core tooling ( Ewels *et al.*, 2020) and MultiQC ( Ewels *et al.*, 2016), relying on the Conda package manager, the Bioconda initiative ( Grüning *et al.*, 2018), the Biocontainers infrastructure ( da Veiga Leprevost *et al.*, 2017), as well as the Docker ( Merkel, 2014) and Singularity ( Kurtzer *et al.*, 2017) containerisation solutions.

Table 4 contains a list of relevant software tool versions and sources.

**Table 4.** Software tools: versions and sources.

Software tool	Version	Source
BEDTools	2.30.0	https://github.com/arq5x/bedtools2
BLAST	2.14.0	ftp://ftp.ncbi.nlm.nih.gov/blast/executables/blast+/
BlobToolKit	4.3.9	https://github.com/blobtoolkit/blobtoolkit
BUSCO	5.5.0	https://gitlab.com/ezlab/busco
bwa-mem2	2.2.1	https://github.com/bwa-mem2/bwa-mem2
Cooler	0.8.11	https://github.com/open2c/cooler
DIAMOND	2.1.8	https://github.com/bbuchfink/diamond
fasta_windows	0.2.4	https://github.com/tolkit/fasta_windows
FastK	1.1	https://github.com/thegenemyers/FASTK
Gfastats	1.3.6	https://github.com/vgl-hub/gfastats
GoaT CLI	0.2.5	https://github.com/genomehubs/goat-cli
Hifiasm	0.16.1	https://github.com/chhylp123/hifiasm
HiGlass	1.13.4	https://github.com/higlass/higlass
Merqury.FK	1.1.2	https://github.com/thegenemyers/MERQURY.FK
Minimap2	2.24-r1122	https://github.com/lh3/minimap2
MitoHiFi	3	https://github.com/marcelauliano/MitoHiFi
MultiQC	1.14, 1.17, and 1.18	https://github.com/MultiQC/MultiQC
NCBI Datasets	15.12.0	https://github.com/ncbi/datasets
Nextflow	23.04.1	https://github.com/nextflow-io/nextflow
PretextView	0.2.5	https://github.com/sanger-tol/PretextView
purge_dups	1.2.5	https://github.com/dfguan/purge_dups
samtools	1.19.2	https://github.com/samtools/samtools
sanger-tol/ascc	-	https://github.com/sanger-tol/ascc
sanger-tol/ blobtoolkit	0.5.1	https://github.com/sanger-tol/blobtoolkit
Seqtk	1.3	https://github.com/lh3/seqtk
Singularity	3.9.0	https://github.com/sylabs/singularity
TreeVal	1.2.0	https://github.com/sanger-tol/treeval
YaHS	1.2a.2	https://github.com/c-zhou/yahs

### Genome annotation

The Ensembl Genebuild annotation system ( Aken *et al.*, 2016) was used to generate annotation for the *Xanthorhoe designata* assembly (GCA_963582015.1) in Ensembl Rapid Release at the EBI. Annotation was created primarily through alignment of transcriptomic data to the genome, with gap filling via protein-to-genome alignments of a select set of proteins from UniProt ( UniProt Consortium, 2019).

### Wellcome Sanger Institute – Legal and Governance

The materials that have contributed to this genome note have been supplied by a Darwin Tree of Life Partner. The submission of materials by a Darwin Tree of Life Partner is subject to the **‘Darwin Tree of Life Project Sampling Code of Practice’**, which can be found in full on the Darwin Tree of Life website here. By agreeing with and signing up to the Sampling Code of Practice, the Darwin Tree of Life Partner agrees they will meet the legal and ethical requirements and standards set out within this document in respect of all samples acquired for, and supplied to, the Darwin Tree of Life Project.

Further, the Wellcome Sanger Institute employs a process whereby due diligence is carried out proportionate to the nature of the materials themselves, and the circumstances under which they have been/are to be collected and provided for use. The purpose of this is to address and mitigate any potential legal and/or ethical implications of receipt and use of the materials as part of the research project, and to ensure that in doing so we align with best practice wherever possible. The overarching areas of consideration are:

•     Ethical review of provenance and sourcing of the material

•     Legality of collection, transfer and use (national and international)

Each transfer of samples is further undertaken according to a Research Collaboration Agreement or Material Transfer Agreement entered into by the Darwin Tree of Life Partner, Genome Research Limited (operating as the Wellcome Sanger Institute), and in some circumstances other Darwin Tree of Life collaborators.

## Data Availability

European Nucleotide Archive: Xanthorhoe designata. Accession number PRJEB63491; https://identifiers.org/ena.embl/PRJEB63491. The genome sequence is released openly for reuse. The *Xanthorhoe designata* genome sequencing initiative is part of the Darwin Tree of Life (DToL) project. All raw sequence data and the assembly have been deposited in INSDC databases. Raw data and assembly accession identifiers are reported in Table 1 and Table 2. Production code used in genome assembly at the WSI Tree of Life is available at https://github.com/sanger-tol.

## Author information

Members of the Natural History Museum Genome Acquisition Lab are listed here: https://doi.org/10.5281/zenodo.12159242.

Members of the Darwin Tree of Life Barcoding collective are listed here: https://doi.org/10.5281/zenodo.12158331.

Members of the Wellcome Sanger Institute Tree of Life Management, Samples and Laboratory team are listed here: https://doi.org/10.5281/zenodo.12162482.

Members of Wellcome Sanger Institute Scientific Operations: Sequencing Operations are listed here: https://doi.org/10.5281/zenodo.12165051.

Members of the Wellcome Sanger Institute Tree of Life Core Informatics team are listed here: https://doi.org/10.5281/zenodo.12160324.

Members of the Tree of Life Core Informatics collective are listed here: https://doi.org/10.5281/zenodo.12205391.

Members of the Darwin Tree of Life Consortium are listed here: https://doi.org/10.5281/zenodo.4783558.
