# Supplementary material for: The genome sequence of the Flame Carpet moth, Xanthorhoe designata (Hufnagel, 1767) (Lepidoptera: Geometridae)
Source: Wellcome Open Res. 2025 Oct 1;10:110. Originally published 2025 Feb 28. [Version 2] doi: 10.12688/wellcomeopenres.23757.2 (PMC12511866; doi:10.12688/wellcomeopenres.23757.2)
Supplement: Peer review history

# Reviewer report: Reviewer response for version 2

**DOI:** 10.21956/wellcomeopenres.27577.r134807

**Published:** 2025-10-09

**Authors:** Christopher B. Cunningham

**Affiliations:** 1 University of Georgia, Georgia, USA

The revisions addressed the comments.

Are sufficient details of methods and materials provided to allow replication by others?

Partly

Is the rationale for creating the dataset(s) clearly described?

No

Are the datasets clearly presented in a useable and accessible format?

Yes

Are the protocols appropriate and is the work technically sound?

Yes

Reviewer Expertise:

Insects, Behavior, Genetics, Genomics, Epigenetics.

I confirm that I have read this submission and believe that I have an appropriate level of expertise to confirm that it is of an acceptable scientific standard.

# Reviewer report: Reviewer response for version 2

**DOI:** 10.21956/wellcomeopenres.27577.r134808

**Published:** 2025-10-03

**Authors:** Frederique Hilliou

**Affiliations:** 1 Institut Sophia Agrobiotech, Universite Cote d'Azur, INRAE, Nice, Provence-Alpes-Côte d'Azur, France

Genes annotation of this genome is now available at:

  https://ftp.ebi.ac.uk/pub/ensemblorganisms/Xanthorhoe_designata/GCA_963582015.1/ensembl/geneset/2024_04/

Are sufficient details of methods and materials provided to allow replication by others?

Yes

Is the rationale for creating the dataset(s) clearly described?

Yes

Are the datasets clearly presented in a useable and accessible format?

Yes

Are the protocols appropriate and is the work technically sound?

Yes

Reviewer Expertise:

NA

I confirm that I have read this submission and believe that I have an appropriate level of expertise to confirm that it is of an acceptable scientific standard.

# Reviewer report: Reviewer response for version 1

**DOI:** 10.21956/wellcomeopenres.26205.r119901

**Published:** 2025-03-26

**Authors:** Frederique Hilliou

**Affiliations:** 1 Institut Sophia Agrobiotech, Universite Cote d'Azur, INRAE, Nice, Provence-Alpes-Côte d'Azur, France

The manuscript presents the genome assembly from a female Xanthorhoe designata

 (Flame Carpet; Arthropoda; Insecta; Lepidoptera; Geometridae). It provides a clear presentation of the sample used to obtain the genome as well as all the technical details used from the DNA extraction to the bioinformatic protocols used to assemble the genome.

 I was able to access all the data except the annotation of the genome at EBI. Maybe it will be only available after an approved submission? 

 The genome was “Annotated at EBI with Assemble Rapid Released”: could you please explain what it means?*

**Genome annotation:**

 The Ensembl Genebuild annotation system ( Aken *et al.*, 2016) was used to generate annotation for the Xanthorhoe designate assembly (GCA_963582015.1) in Ensembl Rapid Release at the EBI. Annotation was created primarily through alignment of transcriptomic data to the genome, with gap filling via protein-to-genome alignments of a select set of proteins from UniProt ( UniProt Consortium, 2019).

 I was not able to access Ensembl Genebuild but many details of the annotation process are given in Aken *et al.,* 2016

 “The assembly was decontaminated using the Assembly Screen for Cobionts and Contaminants (ASCC) pipeline (article in preparation).”: Can some more details be added in this manuscript before the indexing?

 Overall this manuscript provides information on this new genome of the Geometridae family that I believe will be very useful for insect genomic research community as well as to research involving comparative genomic.

Are sufficient details of methods and materials provided to allow replication by others?

Yes

Is the rationale for creating the dataset(s) clearly described?

Yes

Are the datasets clearly presented in a useable and accessible format?

Yes

Are the protocols appropriate and is the work technically sound?

Yes

Reviewer Expertise:

NA

I confirm that I have read this submission and believe that I have an appropriate level of expertise to confirm that it is of an acceptable scientific standard.

# Reviewer report: Reviewer response for version 1

**DOI:** 10.21956/wellcomeopenres.26205.r119904

**Published:** 2025-03-19

**Authors:** Christopher B. Cunningham

**Affiliations:** 1 University of Georgia, Georgia, USA

I think this is a good resource for the insect genomics community. The methods are sound and relatively well documented. The article needs a few more details to be reasonably reproducible.

**Abstract.** There is no justification within the abstract, just results. At least a sentence that this was part of a large survey of insects is needed. And one sentence for its possible value. Also, no estimates of the genome (% assembled of estimated size) or its gene compliment completeness (BUSCO of genome and annotation) are given.

**Keywords.** Should be alphabetical order. Nothing from the title needs to be repeated.   

**Background.** Almost all the details of its life history are irrelevant to the reader. The paper is not about its ecology or morphology, but about its genetics. The reader does not really understand that from this background. Are there close relatives that have been sequenced? Does it serve as a model for any physiology. Direct mention the motivation is that it is part of the Darwin Tree of Life project.

 Figure 2/3/4/5 titles and description in manuscript. The paragraph of Fig 2/3 should say the results, not what the figure contains – that should be found in the caption. The titles should be informative and tell the reader what you would like them to understand about the data; e.g., Fig 3. Little contamination was found in the final genome assembly.

 Figure 4. has very little information beyond what is found elsewhere in the manuscript and can easily be summarized as a sentence in the results. The same is true of Fig 5

 BUSCO completeness needs to be assessed with the updated OrthoDB version 12. Difference from 10 to 12 have made a huge difference for several insects I work with and colleagues have reported the same. This is necessary for the work to be moved to indexing. The genome annotation also needs an estimate of completeness of expected genes. 

 All parameter setting if they were different from default of each piece of bioinformatic software needs to be specified. Just put a sentence to begin that everything was default unless specified otherwise. What assessment was done to ensure that default was adequate?

 Table 4 is of little added value. Just add the version numbers of software used inline at the appropriate places in the methods section.

 Is the Ethics statement needed? It just says the provider of the sample should meet some agreed upon standard, but does not actually say that they did in this case. Either drop the statement or actually say the standard was met for this sample. This is not a paper about the standards of collection.

 Add something to the report that contextualize the research for the reader. Is this species the first of a taxon to be assembled, is it an outgroup to some established model species, is it now possible to investigate some interesting aspect of the species biology, etc? This does not to be extensive or highly directed, but there is currently no justification of this work outside of the Data Availability Statement. Even Data Note should contain rationale for the work.

Are sufficient details of methods and materials provided to allow replication by others?

Partly

Is the rationale for creating the dataset(s) clearly described?

No

Are the datasets clearly presented in a useable and accessible format?

Yes

Are the protocols appropriate and is the work technically sound?

Yes

Reviewer Expertise:

Insects, Behavior, Genetics, Genomics, Epigenetics.

I confirm that I have read this submission and believe that I have an appropriate level of expertise to state that I do not consider it to be of an acceptable scientific standard, for reasons outlined above.
